# Characterization of Genomic Alterations in Radiation-Associated Breast Cancer among Childhood Cancer Survivors, Using Comparative Genomic Hybridization (CGH) Arrays

**DOI:** 10.1371/journal.pone.0116078

**Published:** 2015-03-12

**Authors:** Xiaohong R. Yang, J. Keith Killian, Sue Hammond, Laura S. Burke, Hunter Bennett, Yonghong Wang, Sean R. Davis, Louise C. Strong, Joseph Neglia, Marilyn Stovall, Rita E. Weathers, Leslie L. Robison, Smita Bhatia, Kiyohiko Mabuchi, Peter D. Inskip, Paul Meltzer

**Affiliations:** 1 Division of Cancer Epidemiology & Genetics, National Cancer Institute, National Institutes of Health, Bethesda, Maryland, United States of America; 2 Center of Cancer Research, National Cancer Institute, National Institutes of Health, Bethesda, Maryland, United States of America; 3 Department of Laboratory Medicine and Pathology, Children's Hospital and Ohio State University College of Medicine, Columbus, Ohio, United States of America; 4 Department of Genetics, The University of Texas MD Anderson Cancer Center, Houston, Texas, United States of America; 5 Department of Pediatrics, University of Minnesota School of Medicine, Minneapolis, Minnesota, United States of America; 6 Department of Radiation Physics, The University of Texas MD Anderson Cancer Center, Houston, Texas, United States of America; 7 Epidemiology and Cancer Control, St. Jude Children's Research Hospital, Memphis, Tennessee, United States of America; 8 Department of Pediatrics, University of Alabama at Birmingham, Birmingham, Alabama, United States of America; The University of Hong Kong, CHINA

## Abstract

Ionizing radiation is an established risk factor for breast cancer. Epidemiologic studies of radiation-exposed cohorts have been primarily descriptive; molecular events responsible for the development of radiation-associated breast cancer have not been elucidated. In this study, we used array comparative genomic hybridization (array-CGH) to characterize genome-wide copy number changes in breast tumors collected in the Childhood Cancer Survivor Study (CCSS). Array-CGH data were obtained from 32 cases who developed a second primary breast cancer following chest irradiation at early ages for the treatment of their first cancers, mostly Hodgkin lymphoma. The majority of these cases developed breast cancer before age 45 (91%, n = 29), had invasive ductal tumors (81%, n = 26), estrogen receptor (ER)-positive staining (68%, n = 19 out of 28), and high proliferation as indicated by high Ki-67 staining (77%, n = 17 out of 22). Genomic regions with low-copy number gains and losses and high-level amplifications were similar to what has been reported in sporadic breast tumors, however, the frequency of amplifications of the 17q12 region containing human epidermal growth factor receptor 2 (HER2) was much higher among CCSS cases (38%, n = 12). Our findings suggest that second primary breast cancers in CCSS were enriched for an “amplifier” genomic subgroup with highly proliferative breast tumors. Future investigation in a larger irradiated cohort will be needed to confirm our findings.

## Introduction

Ionizing radiation is an established risk factor for breast cancer, and risk increases linearly with dose [1]. Breast cancer is among the most radiogenic tumors identified so far among the atomic-bomb survivors [2]. The greatest relative risk related to radiation exposure was observed for breast cancer among women who were exposed at a young age [3,4]. Similarly, breast cancer is the second most common primary cancer among childhood cancer survivors, following only basal cell carcinoma of the skin [5]. In the survivors, the odds ratio for breast cancer increased linearly with radiation dose, and breast cancer was diagnosed at young ages (median, 35.9 years; range, 20.9 to 49.6 years) [6]. A recent analysis of 1,200 women participating in the Childhood Cancer Survivor Study (CCSS) showed that 25% of those who received >20 gray (Gy) to the chest area developed breast cancer by age 50; among women who received lower doses of radiation (10–19 Gy), 7% developed breast cancer by age 40, versus a less than 2% chance of developing breast cancer by age 50 in the general population [7].

Radiation-associated breast carcinogenesis appears to be a highly complex phenomenon and likely involves accumulating genetic and epigenetic changes. In a recent study characterizing copy number alteration (CNA) and expression profiles in 2,000 breast tumors, Curtis et al. showed that CNAs were associated with profound changes in gene expression through both *cis*- and *trans*-effects [8]. The joint clustering of gene expression and CNA profiles revealed novel breast cancer subtypes that refined previously identified molecular subtypes defined by expression-only profiling. These findings suggest that identifying CNA regions may provide a powerful tool to investigate the molecular basis of radiation-associated breast cancer.

Epidemiologic studies of radiation-exposed cohorts have been primarily descriptive. Molecular events responsible for the development of radiation-associated breast cancer are largely unknown, although recent studies demonstrated that radiation-associated breast tumors were characterized by a high degree of proliferation, high frequency of gene amplifications, in particular HER2 amplification, and enriched with basal-like tumors [9–11]. In this study, we used comparative genomic hybridization arrays (array-CGH) to characterize the CNA profile in breast tumor tissues collected from CCSS cases, the majority of whose breast cancer were radiation related [6], to identify possible distinct genomic aberrations related to radiation exposure.

## Methods

### Study population

Data and biologic specimens for the current analysis came from the CCSS, a retrospective cohort study of 14,135 five-year survivors of childhood cancer (age at diagnosis < 21 years) who were diagnosed and treated at any of 26 collaborating institutions in the United States or Canada between January 1, 1970 and December 31, 1986. Eligible childhood cancer diagnoses included leukemia, central nervous system cancer, Hodgkin lymphoma (HL), non-Hodgkin lymphoma (NHL), renal cancer, neuroblastoma, soft tissue sarcoma or bone sarcoma. The overall study design and characteristics of the cohort were described in detail previously [12]. Patients included in the present study were CCSS cases included in a previous analysis of breast cancer risk [6] and additional cases diagnosed subsequent to that study. The CCSS study was approved by human subjects review committees at each participating institution. All study participants provided informed consent. The current study of using de-identified archived tumor tissue was exempted from review by the National Institutes of Health Office of Human Subject Research.

### Radiation dosimetry

Radiation dose to the presumed site of origin of the breast cancer was estimated for the previous study [6] by medical physicists using radiotherapy records collected for the CCSS cohort [13]. Detailed tumor site-specific dosimetry was not available for the newly identified breast cancer cases. For consistency, we report maximal chest dosage for all cases.

### Pathology review

Most of the breast cancers in the CCSS cohort were self-reported by the participants via a periodic questionnaire. A small minority of cases were first discovered from other medical records being collected. Following self-report, an investigation was made to obtain the pathology report and to request the participant’s permission to use paraffin material from their tumor for research. Immunohistochemical (IHC) staining of estrogen receptor (ER) and Ki-67 were performed in a diagnostic pathology laboratory using a Ventana autostainer (Ventana Medical Systems, Inc., Tucson, AZ). The cutoff for Ki-67 low versus high proliferative index was positive staining of 10% cancer cell nuclei.

### Array-CGH

Archival formalin-fixed paraffin-embedded (FFPE) tumor blocks were identified from 49 breast cancer cases (41 invasive and 8 *in situ*) in the CCSS cohort. Ten 5-μm unstained tumor sections were used for DNA extraction with enrichment for tumor cells by micro-dissection. Sufficient DNA (1 μg) was obtained from 38 cases. Test and reference DNA (from Promega, Madison, WI) were labeled with Cy5 and Cy3, respectively, and co-hybridized to Agilent CGH arrays containing 180,000 probes tiled across the genome. The intensity of the two fluorescent dyes that reflects chromosomal imbalances between test and reference samples was extracted using Agilent Feature-Extraction software. The log2 ratio was then created for each probe and was used as input to Nexus Copy Number (Biodiscovery, Hawthorne, CA). The Rank Segmentation algorithm (significant threshold = 1 x 10^–6^) was used to identify copy number alterations at the probe level. Low-level copy number gains and losses were defined as absolute log2 ratios larger than 0.3. Amplifications were defined as log2 ratios larger than 1. For example, human epidermal growth factor receptor 2 (HER2, ERBB2) amplification was defined as log2 ratios > 1 for at least 5 consecutive probes in the 17q12 region containing the HER2 gene. Both objective and subjective measures of data quality were used. Among 38 tumors analyzed, 6 had low quality array-CGH data, mostly because of low signal-to-noise ratio (Nexus quality score > 0.3 and/or by visualization), and were removed from further analysis (high quality scores suggest elevated noise to signal ratio). The average quality score of remaining 32 samples was 0.14 (range: 0.06–0.28), which was within the acceptable range recommended by Nexus Copy Number.

### Statistical analysis

Differences between chest radiation exposure with regard to CGH profile (amplifier vs. non-amplifier), ER status, tumor grade, and Ki-67 status were examined using the chi-square test. The Fisher’s exact test was used when expected number of samples in any comparison group was less than 5.

## Results

In total, array-CGH data was obtained from 32 CCSS cases whose characteristics are shown in Tables 1 and 2. The majority of these cases (N = 21) had HL as the first cancer and the other 11 cases had NHL, bone cancer, leukemia, soft tissue sarcoma, or kidney cancer. Actual dose to the site where the breast cancer developed and systemic chemotherapy doses are available for most of these cases (30 for radiation treatment (RT) and 32 for chemotherapy). Among 30 cases with known radiation status, 21 received radiation directly to the chest (up to 57 Gy), 1 case had RT, but not to the chest or an adjacent body region, 3 cases had RT that included arm, neck or abdomen but not chest, and 5 cases had no RT. Age at diagnosis of the first cancer for all CCSS cases in this study was between 12 and 20 years; therefore, age at radiation exposure was young for all members of this cohort. Among these cases, 26 were invasive ductal carcinoma, two were invasive lobular carcinoma, three were ductal carcinoma *in situ* (DCIS), and one had both *in-situ* ductal and *in-situ* lobular carcinoma. Most of these cases (N = 29) had early-onset breast cancer (before age 45), invasive ductal tumors (N = 26), ER-positive staining (N = 16 of 25 invasive, 3 of 3 for *in situ*), and high proliferation as indicated by high Ki-67 staining (N = 16 of 19 invasive, 1 of 3 *in situ*).

**Table 1:** Patient characteristics and treatment of CCSS cases included in this study.

Subject	Type 1st cancer	Age at 1st cancer	Chemo	Chest RT	MaxChest dosage (Gy)^a^
CCSS1	HL	13	No	Yes	45
CCSS2	HL	12	No	Yes	46
CCSS3	HL	15	No	Yes	44
CCSS4	HL	17	No	Yes	50
CCSS5	HL	18	Yes	Yes	45
CCSS6	HL	19	Yes	Yes	45
CCSS7	HL	9	Yes	No	SL
CCSS8	HL	10	No	Yes	35
CCSS9	HL	19	No	Yes	44
CCSS10	HL	18	No	UNK	UNK
CCSS11	HL	20	Yes	No	SH
CCSS12	HL	18	Yes	Yes	42
CCSS13	HL	14	No	Yes	41
CCSS14	HL	14	No	Yes	44
CCSS15	HL	15	No	Yes	44
CCSS16	HL	15	No	Yes	51
CCSS17	HL	10	No	Yes	45
CCSS18	HL	15	No	No	0
CCSS19	HL	14	Yes	Yes	35
CCSS20	HL	20	Yes	Yes	38
CCSS21	HL	19	No	Yes	41
CCSS22	NHL	20	Yes	No	SH
CCSS23	Bone cancer	16	Yes	No	SH
CCSS24	Kidney(Wilms)	15	Yes	Yes	18
CCSS25	Kidney(Wilms)	10	Yes	Yes	14
CCSS26	Bone cancer	16	No	Yes	15
CCSS27	Leukemia	14	Yes	No	0
CCSS28	Leukemia	15	Yes	No	0
CCSS29	Bone cancer	13	No	No	0
CCSS30	NHL	17	Yes	UNK	UNK
CCSS31	Soft tissue sarcoma	13	Yes	Yes	32
CCSS32	Kidney(Wilms)	15	Yes	No	0

Abbreviations: HL = Hodgkin lymphoma, NHL = non-Hodgkin lymphoma, RT = radiotherapy, UNK = unknown.

^a^0 = no direct treatment to the chest, UNK = unknown dose, >0 and < 70 Gy = direct treatment to the chest, SL = (low scatter) patient had RT, but not to the chest or an adjacent body region, SH = (high scatter) patient had RT that included arm, neck or abdomen but not chest.

**Table 2:** Breast tumor characteristics of CCSS cases included in this study.

Subject	Age at BC	Type BC	Histology	Grade^a^	ER	Ki-67
CCSS1	27	Invasive	Ductal	UNK	Negative	High
CCSS2	39	Invasive	Ductal	Intermediate	Positive	Low
CCSS3	38	Invasive	Ductal	High	Negative	High
CCSS4	35	In Situ	DCIS+LCIS	Low	Positive	Low
CCSS5	38	Invasive	Ductal	High	Negative	High
CCSS6	39	Invasive	Ductal	Intermediate	Positive	High
CCSS7	30	Invasive	Ductal	High	Positive	High
CCSS8	31	Invasive	Ductal	Low	Positive	Low
CCSS9	49	Invasive	Invasive locular+DCIS	UNK	Positive	High
CCSS10	39	In Situ	DCIS	High	Positive	Low
CCSS11	38	Invasive	Ductal	High	ND	High
CCSS12	44	Invasive	Ductal	High	Negative	High
CCSS13	36	Invasive	Ductal	Intermediate	Positive	High
CCSS14	45	Invasive	Ductal	High	Negative	High
CCSS15	36	Invasive	Lobular	Intermediate	Positive	High
CCSS16	38	Invasive	Ductal	Intermediate	Positive	ND
CCSS17	37	In Situ	DCIS	Intermediate	Positive	High
CCSS18	42	Invasive	Ductal	High	Negative	High
CCSS19	40	Invasive	Ductal	High	Positive	High
CCSS20	48	Invasive	Ductal	High	Positive	High
CCSS21	40	Invasive	Ductal	High	Positive	High
CCSS22	42	Invasive	Ductal	Intermediate	Negative	High
CCSS23	42	Invasive	Ductal	Intermediate	Positive	ND
CCSS24	40	In Situ	DCIS	High	ND	ND
CCSS25	37	Invasive	Ductal	UNK	ND	ND
CCSS26	42	Invasive	Ductal	Intermediate	Positive	ND
CCSS27	40	Invasive	Ductal	High	Negative	ND
CCSS28	48	Invasive	Ductal	Low	Positive	ND
CCSS29	40	Invasive	Ductal	UNK	Positive	ND
CCSS30	42	Invasive	Ductal	Low	Positive	Low
CCSS31	42	Invasive	Ductal	UNK	ND	ND
CCSS32	37	Invasive	Ductal	High	Negative	ND

Abbreviations: BC = breast cancer, DCIS = ductal cell in situ, ER = estrogen receptor, ND = not determined, UNK = unknown.

^a^Grade: low = well differentiated or grade I, intermediate = moderately differentiated or grade II, high = poorly differentiated or grade III.

**Fig 1:**
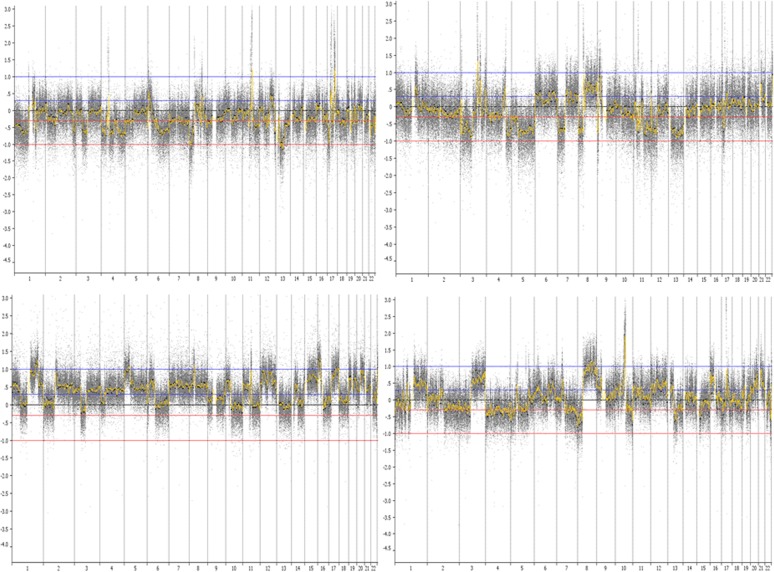
Array-CGH images of CCSS cases displaying “complex-amplifier” genomic profiles. Test and reference DNA were labeled with different dyes and co-hybridized to Agilent CGH arrays containing 180,000 probes tiled across the genome. The chromosome number is shown at the bottom of the figure. Y axis shows the log2 ratios. Low-level copy number gains and losses were defined as absolute log2 ratios larger than 0.3. Amplifications were defined as log2 ratios larger than 1.

Table 3 shows the CNA profile for each case, listing the most common amplified genes or regions and low-level copy number changes involving whole or parts of chromosomes. A detailed list of CNAs is shown in Table A in S1 File. Among the 28 invasive cases with array-CGH data, 5 cases showed “simple” genomic changes characterized by very few copy number changes other than gain of 1q and loss of 16q; 5 cases displayed a more “complex” profile with extensive low-copy number changes but with no high-level amplifications. The majority of CCSS cases (N = 18) displayed amplifications in multiple regions (Table 3, Table A in S1 File). Among the amplifiers, the majority (N = 16) showed a “complex-amplifier” profile which is characterized by the presence of both low copy number changes and high-level amplifications (Fig. 1). The four *in situ* cases showed similar CNA profiles (1 simple, 3 amplifier), and we combined them with invasive cases in subsequent analyses. The most frequent low-copy number changes were the gains of 1q, 3q, 6p, 8q, 16p, and 17q, and losses of 3p, 6q, 8p, 9p, 10q, 11q, 16q and 17p. The most frequent amplifications were 17q12 (containing HER2, n = 12 [38%]), 17q21–24 (n = 10 [31%]), 11q13 (containing CCND1, n = 7 [22%]), 8p11.2 (n = 7 [22%]), 8p12 (containing FGFR1, n = 5 [16%]), and 8q24 (containing MYC, n = 5 [16%]). Among 7 cases with HER2 IHC data available, results from IHC and arrayCGH were consistent for all but one sample (Table 3).

**Table 3:** ArrayCGH profile and molecular subtypes of CCSS cases included in this study.

Subject	CGH subtype^a^	Recurrent CNAs	Subtype^b^
CCSS1	Amplifier	HER2 amp, CCND1 amp, 1p loss, 3p loss, 5p loss, 6q loss, 8p loss, 9p loss, 17p loss	ER-HER2+
CCSS2	Amplifier	CCND1 amp, 16p gain, 8p loss, 16q loss	Luminal
CCSS3	Amplifier	8p11.2amp, 8q21–23amp, 12q12amp, 19q12–13.2amp, 10q loss	ER-HER2-^c^
CCSS4	Simple	16q loss	Luminal
CCSS5	Amplifier	FGFR1 amp, 8q gain, 3p loss, 8p loss,17p loss	ER-HER2-
CCSS6	Amplifier	FGFR1 amp, CCND1 amp, 1q gain, 6p gain, 16p gain	Luminal
CCSS7	Amplifier	HER2 amp, ZNF217 amp, 1q gain, 8q gain, 16p gain, 3p loss, 8p loss	ER+HER2+^c^
CCSS8	Amplifier	MYC amp, ZNF217 amp, 16p gain, 3p loss, 16q loss	Luminal
CCSS9	Amplifier	HER2 amp, 6q loss, 16q loss	ER+HER2+^c^
CCSS10	Amplifier	CCND1 amp, 1q gain, 8q gain, 16p gain, 6q loss, 8p loss, 17p loss	Luminal
CCSS11	Simple	1q gain, 3q gain, 10q gain	ND
CCSS12	Amplifier	CCND1 amp, 16p gain	ER-HER2-^c^
CCSS13	Simple	16q loss	Luminal
CCSS14	Amplifier	HER2 amp, 1q gain, 6q loss, 8p loss, 9p loss, 10q loss	ER-HER2+
CCSS15	Complex	1q gain, 6p gain, 17q gain, 3p loss, 6q loss, 13q loss, 17p loss	Luminal
CCSS16	Amplifier	MYC amp, FGFR1 amp, 10q loss, 16q loss	Luminal
CCSS17	Amplifier	HER2 amp	ER+HER2+
CCSS18	Amplifier	HER2 amp, 3q gain, 3p loss, 9p loss	ER-HER2+
CCSS19	Complex	1q gain, 17q gain, 1p loss, 6q loss, 13q loss, 16q loss, 17p loss	Luminal
CCSS20	Simple	1q gain, 18q loss	Luminal^c^
CCSS21	Amplifier	HER2 amp, CCND1 amp, FGFR1 amp, MYC amp, 8q gain, 16p gain, 8p loss, 16q loss, 17p loss	ER+HER2+
CCSS22	Amplifier	HER2 amp	ER-HER2+
CCSS23	Amplifier	CCND1 amp, 1q gain, 16p gain	Luminal
CCSS24	Amplifier	HER2 amp, 9p loss, 10q loss	ND
CCSS25	Amplifier	FGFR1 amp, HER2 amp	ND
CCSS26	Simple	1p loss, 10q loss	Luminal^c^
CCSS27	Amplifier	HER2 amp, MYC amp, ZNF217 amp, 5p gain, 8q gain, 9q gain, 20q gain, 8p loss	ER-HER2+^c^
CCSS28	Simple	8q gain	Luminal
CCSS29	Complex	3q gain, 8q gain, 9q gain, 17q gain, 1p loss, 3p loss, 6q loss, 9p loss, 10q loss, 16q loss	Luminal
CCSS30	Complex	10p gain, 16p gain, 20p gain, 10q loss, 16q loss	Luminal
CCSS31	Complex	1q gain, 1p loss, 3p loss, 6q loss, 9p loss, 11q loss	ND
CCSS32	Amplifier	HER2 amp, MYC amp, 8p loss, 9p loss, 17p loss, 11p gain, 17q gain	ER-HER2+

^a^Array-CGH subtype: simple: very few copy number changes other than gain of 1q and loss of 16q; complex; extensive low copy number changes but with no high-level amplifications; amplifiers: high-level amplifications usually accompanied by low copy number changes.

^b^ER positivity was determined by immunohistochemistry; HER2 positivity was defined by the presence of amplification of 17q12 region (log2 ratio>1) based on aCGH data.

^c^HER2 IHC status was available. IHC and CGH data were concordant for all but one sample (CCSS20), for which IHC was positive and amplification was negative.

Among 28 cases with ER status available, 15 cases (54%) had luminal tumors, defined as positive for ER by IHC and negative for HER2 amplification; 4 cases (14%) were double positive (ER+ and HER2amp); 6 cases (21%) were ER- and HER2amp; and 3 cases (11%) were double negative (ER-negative and HER2amp-negative) tumors. The frequency of high-level amplifications was significantly lower in ER+ tumors (53% vs. 100% in ER-, p = 0.01). The difference remained significant when taking out the four in situ cases. All nine cases with a simple or complex CGH profile with the absence of amplifications had luminal tumors (ER status was undetermined in one “simple” and one “complex” case). The remaining six luminal tumors were amplifiers, harboring both amplifications (mostly in CCND1) and low-copy changes such as 16p gain and 16q loss. Among ten HER2-amplified tumors with known ER status, ER+ (N = 4) and ER- tumors (N = 6) showed similar CNAs in other chromosome regions. The three cases with double-negative tumors all had “complex-amplifier” CNA profiles.

Copy number changes did not appear to vary by whether the patient received chemotherapy in addition to radiation treatment or not.

## Discussion

In this study, we characterized molecular and genetic changes in tumor tissue collected from CCSS breast cancer cases, the majority of whom were treated with chest radiation for a prior tumor when they were under 20 years of age. We found that breast cancers in the majority of these cases were positive for ER (68%, n = 19 out of 22), highly proliferative as indicated by Ki-67 staining (77%, n = 17 out of 22), and frequently had high-level amplifications (66%). The frequency of HER2 amplification appeared to be particularly higher in this irradiated series of cases (38%, n = 12) compared to breast cancer among young women in the general population [14–17].

Genomic regions with low-copy gains and losses and high-level gene amplifications among CCSS cases were similar to those reported for breast cancers in the general population, with the most frequent gains on chromosomes 1q, 3q, 6p, 8q, 16p, and 17q; losses on 3p, 6q, 8p, 9p, 10q, 11q, 16q, and 17p; and high-level amplifications of 8p 11.2–12, 8q24, 11q13, 17q12, and 17q21–24. Previous array-CGH studies classified breast tumors into three genomic subtypes (simplex, complex, and amplifier) and found a significant correlation between these genomic subtypes and molecular subtypes defined by gene expression profiles [18,19]. Using a similar classification scheme, we found that a majority of CCSS cases demonstrated a “complex-amplifier” profile, characterized by the presence of extensive low copy number changes and recurrent amplifications. High-level amplifications have been associated with short telomere length, indicating high genomic instability, and poor prognosis independent of tumor grade and nodal status [18,19]. The majority of the CCSS cases (17 out of 22) in our study demonstrated proliferation as indicated by Ki-67 staining, suggesting that radiation may induce a highly proliferative subtype of breast cancer through gene amplifications. It is possible that the high frequency of amplifications may be a characteristic of young breast cancer cases instead of being driven by radiation exposure since breast cancers in CCSS survivors occurred at young ages. However, the frequency of HER2 amplification in CCSS cases (38%) was still higher compared to young breast cancer (<45 years) in the general population (16%−25%) based on a literature search [14–17]. In addition, we compared the frequency of the most common amplifications to those in breast cancer cases diagnosed before 45 years of age in The Cancer Genome Atlas (TCGA) Research Network (http://cancergenome.nih.gov/) [20] as well as in three published studies with accessible age and array-CGH data [21–23], and we found that the frequency of most amplifications, in particular, regions containing HER2 and possibly CCND1, still appeared higher compared to breast cancer in the general population (Table B in S1 File). Since the vast majority of cases in our study received high-dose radiation and very few cases had no radiation or radiation in other body areas, our study did not have the power in evaluating the radiation dosage in relation to HER2 amplification frequency. However, our data are consistent with the high frequency of HER2 amplification observed in breast cancers among atomic-bomb survivors [10,24], supporting the view that HER2 amplification may be an important mechanism in radiation-associated breast carcinogenesis.

A gene expression profiling analysis of breast tumors from 22 patients who developed breast cancer after HL suggested that radiation-associated tumors were associated with a higher frequency of basal-like tumors [9]. Similarly, a more recent study found that breast cancer patients after radiation therapy were more likely to have triple-negative tumors [11]. Using immunohistochemical ER status and HER2 amplification, we classified breast cancers into four subtypes, luminal, ER+/HER2+, ER-HER2+, and double negative. The frequency of ER+ tumors in our study (68%, n = 19 out of 28) is slightly higher compared to breast cancer among young cases in the general population (50–60%) [15,17], but is consistent with what was reported by Broeks et al. in the study of breast cancer after HL [9]. However, in contrast to the enrichment of basal-like or triple-negative tumors observed in previous studies, our results showed that breast tumors from CCSS cases were preferentially of two subtypes: luminal or HER2+, which is in line with what was observed by Castiglioni in breast tumors developed in women irradiated for HL within 4 years of menarche [25]. Our data is also consistent with findings from previous reports that radiation-associated breast cancers were characterized by more proliferative and aggressive features [9,24]. Since each individual study is unavoidably limited by the small sample size, a collaborative effort combining data from multiple such studies is needed to more accurately characterize the distribution of molecular subtypes in radiation-associated breast tumors.

As expected, all cases without amplifications had luminal tumors in our study; however, luminal tumors also demonstrated extensive heterogeneity in genomic changes. In addition to the simple subtype, 5 luminal cases displayed a complex profile, and the remaining 6 luminal cases harbored high-level amplifications (4 had amplifications in CCND1). Our data are consistent with previous findings that the majority of CCND1-amplified tumors were ER+ [26]. Recently, a large-scale genomic and transcriptomic analysis of 2,000 breast tumors revealed a high-risk 11q13/14 *cis*-acting luminal subtype that was associated with high mortality [8]. This subgroup exhibited high-level amplification in CCND1 as well as alterations in several key cell cycle-related genes, which may be the drivers for the development of this tumor subtype and play a role in its aggressiveness. The higher frequency of ER-positive staining and focal amplifications suggest that breast tumors in CCSS cases may be enriched for a high-risk luminal subtype [27].

The major limitations of our study include small sample size, particularly in the non-irradiated group. There were only nine cases without chest radiation and, among them, 4 cases had radiation exposure but not directly to the chest. Therefore, we were not able to conduct a direct “case-control” comparison within the cohort or to model copy number changes against quantitative radiation dosage because of small sample size. However, our study population is unique; most cases had high-dose radiation at early ages and developed breast cancer when they were young. In addition, we characterized genome-wide CNAs as well as ER expression and proliferation status in these cases, which is lacking in the radiation-associated human breast cancer literature.

In conclusion, data from our study are consistent with previous findings that radiation-associated breast cancer might have a distinct pathogenesis, characterized by high frequency of genomic amplifications and high degree of proliferation. Future studies with large numbers of exposed and non-exposed cases are needed to validate these findings to determine the etiologic link between radiation exposure, early age onset, gene amplifications, and highly proliferative breast cancers.

## Supporting Information

S1 File Table A: Copy number changes in breast tumors in 32 CCSS cases. Table B: Frequency of amplifications in HER2, CCND1, FGFR1, and MYC in young breast cancer cases (age < 45 years) in TCGA and published studies.
(DOC)
