# Supplementary material for: Characterization of Genomic Alterations in Radiation-Associated Breast Cancer among Childhood Cancer Survivors, Using Comparative Genomic Hybridization (CGH) Arrays
Source: PLoS One. 2015 Mar 12;10(3):e0116078. doi: 10.1371/journal.pone.0116078 (PMC4357472; doi:10.1371/journal.pone.0116078)
Supplement: S1 File — Table B: Frequency of amplifications in HER2, CCND1, FGFR1, and MYC in young breast cancer cases (age < 45 years) in TCGA and published studies. (DOC) [file pone.0116078.s001.doc]

Table A. Copy number changes in breast tumors in 32 CCSS cases.

| Subject | Gains | | Losses | | Amplifications | | | | | Well known genes in amplified regions | |
| --- | --- | --- | --- | --- | --- | --- | --- | --- | --- | --- | --- |
| CCSS1 |  | | 1p, 3p, 5p, 6q, 8p, 9p, 11q, 17p, 18q, 19q, 20p | | 1p13.2, 4q12-4q13.1, 8p11.21, 8q22.3, 11q13.2-11q13.3, 11q13.4, 11q13.5-11q14.1, 17q11.2, 17q12-17q21.2, 17q23.1-17q24.1 | | | | | CCND1, HER2 | |
| CCSS2 | 16p | | 8p, 11q, 16q | | 10p14-10p13, 10p12.31-10p12.2, 11q13.2-11q13.3, 11q13.5-11q14.1, 11q14.2, 14q13.3, 14q21.2-14q21.3, 14q22.1 | | | | | CCND1 | |
| CCSS3 |  | | 10q | | 8p11.2, 8q21-23, 12q12, 19q12, 19q13.12-19q13.2 | | | | |  | |
| CCSS4 |  | | 16q | |  | | | | |  | |
| CCSS5 | 7q, 8q | | 3p, 7p, 8p, 11q, 12p, 17p | | 3p25.3, 3q21.3-3q22.2, 3q25.1, 3q25.2, 3q25.31, 4q31.22-4q31.23, 8p12-8p11.23, 8p11.22-8p11.21, 9p21.3, 11p13, 11p12, 17p11.2, 17q21.31 | | | | | FGFR1 | |
| CCSS6 | 1q, 2q, 3q, 5p, 6p, 10p, 16p, 17q, 20q | |  | | 8p12-8p11.23, 8p11.21, 11q13.1-11q13.3 | | | | | FGFR1, CCND1 | |
| CCSS7 | 1q, 3q, 8q, 16p | | 3p, 7q, 8p | | 5q11.2, 5q31.3, 7p14.1, 7p13, 8p11.2, 10p11.22, 10q11.21, 10q21.3, 10q22.1-10q22.3, 17q12-17q21.2, 17q22-24, 20q13, 21q21.2-21q21.2 | | | | | HER2, ZNF217 | |
| CCSS8 | 16p | | 3p, 16q | | 3p26.2, 8q24.13-8q24.21, 20q13.2-20q13.32, 20q13.33 | | | | | MYC, ZNF217 | |
| CCSS9 |  | | 6q, 16q | | 6p21.2, 6p21.1, 6p12.3, 17q12, 17q23.1, 17q23.2 | | | | | HER2 | |
| CCSS10 | 1q, 7p, 8q, 16p, 17q | | 6q, 8p, 11q, 17p | | 11q13.1, 11q13.2-11q13.3, 17p11.2 | | | | | CCND1 | |
| CCSS11 | 1q, 3q, 10q | |  | |  | | | | |  | |
| CCSS12 | 10p, 16q | |  | | 8q22.2-8q22.3, 8q23.2, 10q11.22, 11q13.2-11q13.4, 12p13.33, 18q11.2, 20q11.21 | | | | | CCND1 | |
| CCSS13 |  | | 16q | |  | | | | |  | |
| CCSS14 | 1q | | 3p, 6q, 8p, 9p, 10q | | 17q11.2, 17q12-17q21.2, 17q21.32, 17q21.33, 17q22, 17q24.1, 17q24.2, 17q24.3-17q25.1 | | | | | HER2 | |
| CCSS15 | 1q, 6p, 17q | | 3p, 6q, 13q | |  | | | | |  | |
| CCSS16 |  | | 10q, 11q, 16q, 17p | | 8p12-8p11.23, 8q22.2-8q22.3, 8q23.3, 8q24.12-8q24.21 | | | | | FGFR1, MYC | |
| CCSS17 |  | |  | | 8p11.2, 17q12, 17q22-24 | | | | | HER2 | |
| CCSS18 | 3q, 9q | | 3p, 9p | | 1q21.3-1q22, 17q11.2, 17q12, 17q21.33, 17q22, 17q23.1-17q23.2, 17q23.3-17q24.1, 17q24.2, 17q24.3-17q25.1 | | | | | HER2 | |
| CCSS19 | 1q, 17q | | 1p, 6q, 11q, 13q,16q, 17p | |  | | | | |  | |
| CCSS20 | 1q | | 18q | |  | | | | |  | |
| CCSS21 | 8q, 16p, 17q | | 8p, 11q, 16q, 17p | | 5q35.3, 8p12, 8q21-23, 8q24, 11q13.2-11q13.4, 17q12 | | | | | FGFR1, MYC, CCND1, HER2 | |
| CCSS22 |  | |  | | 17q12 | | | | | HER2 | |
| CCSS23 | 1q, 16p | |  | | 11q13.1, 11q13.3 | | | | | CCND1 | |
| CCSS24 |  | | 8q, 9p, 10q | | 12q13.11, 17q12-17q21.1, 17q22, 17q23.1-17q23.2, 17q23.3-17q24.1 | | | | | HER2 | |
| CCSS25 |  | |  | | 8p12, 14q22.1-14q22.2, 17q12, 17q21.31, 17q22-24 | | | | | FGFR1, HER2 | |
| CCSS26 |  | | 1p, 10q | |  | | | | |  | |
| CCSS27 | 5p, 8q, 9q, 10p, 20q | | 8p | | 8p11.2, 8q21-23, 8q24,17q12, 17q22-24, 20q13 | | | | | MYC, HER2, ZNF217 | |
| CCSS28 | 8q | |  | |  | | | | |  | |
| CCSS29 | 3q, 8q, 9q, 17q | | 1p, 3p, 6q, 9p, 10q, 11p, 11q, 12p, 16q, 17p | |  | | | | |  | |
| CCSS30 | 10p, 16p, 20p | | 10q, 16q | |  | | | | |  | |
| CCSS31 | 1q | | 1p, 2p, 3p, 6q, 9p, 11q | |  | | | | |  | |
| CCSS32 | 8q, 9q, 19q | | 8p, 9p, 17p, 19p | | 8q24, 11p15.1, 11p14.3-11p14.2, 17q12, 17q12-17q21.2, 17q21.31, 17q21.32-17q21.33, 17q21.33-17q22, 17q22-17q24.2, 17q25.1 | | | | | MYC, HER2 | |
| Table B. Frequency of amplifications in HER2, CCND1, FGFR1, and MYC in young breast cancer cases (age < 45 years) in TCGA and published studies. | | | | | | | | |  | | |
| **Amplification** | | **CCSS (N=25)** | | **TCGA (N=175)** | | **Andre (N=31)** | **Russnes (N=23)** | **Jonsson G (N=103)** | | |  |
| HER2 | | 40% | | 14% | | 16% | 13% | 9% | | |  |
| CCND1 | | 28% | | 14% | | 16% | 17% | 7% | | |  |
| FGFR1 | | 20% | | 12% | | 13% | 4% | 5% | | |  |
| MYC | | 12% | | 22% | | 23% | 4% | 13% | | |  |
